# Chromosomal microarray in clinical diagnosis: a study of 337 patients with congenital anomalies and developmental delays or intellectual disability

**DOI:** 10.3325/cmj.2017.58.231

**Published:** 2017-06

**Authors:** Ivona Sansović, Ana-Maria Ivankov, Adriana Bobinec, Mijana Kero, Ingeborg Barišić

**Affiliations:** 1 Department of Medical Genetics and Reproductive Health, Children’s Hospital Zagreb, University of Zagreb, School of Medicine, Zagreb, Croatia

## Abstract

**Aim:**

To determine the diagnostic yield and criteria that could help to classify and interpret the copy number variations (CNVs) detected by chromosomal microarray (CMA) technique in patients with congenital and developmental abnormalities including dysmorphia, developmental delay (DD) or intellectual disability (ID), autism spectrum disorders (ASD) and congenital anomalies (CA).

**Method:**

CMA analysis was performed in 337 patients with DD/ID with or without dysmorphism, ASD, and/or CA. In 30 of 337 patients, chromosomal imbalances had previously been detected by classical cytogenetic and molecular cytogenetic methods.

**Results:**

In 73 of 337 patients, clinically relevant variants were detected and better characterized. Most of them were >1 Mb. Variants of unknown clinical significance (VOUS) were discovered in 35 patients. The most common VOUS size category was <300 kb (40.5%). Deletions and *de novo* imbalances were more frequent in pathogenic CNV than in VOUS category. CMA had a high diagnostic yield of 43/307, excluding patients previously detected by other methods.

**Conclusion:**

CMA was valuable in establishing the diagnosis in a high proportion of patients. Criteria for classification and interpretation of CNVs include CNV size and type, mode of inheritance, and genotype-phenotype correlation. Agilent ISCA v2 Human Genome 8x60 K oligonucleotide microarray format proved to be reasonable resolution for clinical use, particularly in the regions that are recommended by the International Standard Cytogenomic Array (ISCA) Consortium and associated with well-established syndromes.

Patients with developmental delay or intellectual disability (DD/ID), autism spectrum disorders (ASD), and congenital anomalies (CA) account for the largest proportion of cytogenetic testing due to their high prevalence in the population. More than 80% of the chromosomal aberrations found in ID/DD or ASD are submicroscopic and not detected by classical cytogenetic methods. Chromosomal microarray (CMA) is used as the first test to detect copy number variations (CNVs) that are major cause of these disorders. Large cohort studies of patients with DD/ID, CA, or ASD screened by microarray found a diagnostic yield of 14–20% compared with ~ 3% for the standard G-banded karyotype (1,2).

The resolution level of the CMA has no limit, ie, it depends on the size and distance between the test probes on the array. Today, there are a number of commercially available array platforms, which differ in genome coverage, resolution, and application. A higher array resolution can mean not only an increased yield of clinical genetic diagnosis, but also a higher level of detection of benign CNVs or variants of unknown clinical significance (VOUS). Therefore, the correct choice of the resolution array platform that balances sensitivity and specificity is very important for clinical practice.

Defining the pathogenicity of CNVs is the major difficulty in the interpretation of the array results. The usual criteria used in the interpretation of the clinical relevance of a CNV are inheritance, size, type, and gene content.

In this study, we present the CMA analysis of 337 patients with DD/ID with or without dysmorphic features, ASD and/or CA. In 30 patients, chromosomal imbalances had been previously detected by classical cytogenetic and molecular cytogenetic methods. These patients were analyzed by CMA to define more precisely the breakpoints and the gene content of the rearrangements and to clarify if there were any additional CNVs. The aim of the study was to determine the diagnostic yield of the CMA analysis on the remaining 307 patients and criteria that could help in classification and interpretation of the CNVs detected.

## Patients and methods

### Patients

The analysis included 337 unrelated patients from Croatia, referred to the Department of Medical Genetics and Reproductive Health, Children's Hospital Zagreb, University of Zagreb, School of Medicine, who were diagnosed by clinical geneticists or pediatricians to have DD/ID, ASD, CA, or a combination of those features. We divided the patients according to the main clinical features into six groups as follows: ID/DD, ASD, CA with/without dysmorphism; ID/DD, CA with/without dysmorphism; ID/DD, ASD with or without dysmorphism; ID/DD with/without dysmorphism; CA with/without dysmorphism; and ASD with/without dysmorphism. The median age of patients at examination was 7 years (range: one months to 25 years). Samples were analyzed by CMA in the period between January 2016 and April 2017. In 30 patients, chromosomal imbalances had already been detected by high resolution conventional cytogenetics, Multiplex Ligation-dependent Probe Amplification (MLPA) or Fluorescence In Situ Hybridization (FISH). To determine the origin of the imbalances, parental blood samples were requested and those available were tested by CMA, conventional cytogenetics or FISH, depending of the size of the imbalances. Informed consent was obtained from all patients or their parents/guardians.

## METHODS

### DNA extraction

Genomic DNA was extracted from peripheral blood lymphocytes using Illustra blood genomicPrep Mini Spin (GE Healthcare Life Sciences, Chalfont St Giles, Buckinghamshire, UK) or NucleoSpin Blood (Macherey-Nagel, Düren, Germany) kit according to the manufacturers’ instructions. DNA concentration and purity were measured using a Qubit 3.0 Fluorometer (Thermo Fisher Scientific, Waltham, MA, USA) and BioDrop uLite Spectrophotometer (Isogen Life Science, De Meern, The Netherlands).

### Chromosomal microarray

High-resolution whole genome analysis was performed using Agilent SurePrint G3 Unrestricted CGH ISCA v2 Human Genome microarrays according to the manufacturers’ instructions (Agilent Technologies, Santa Clara, CA, USA). The 8x60 K oligonucleotide microarray format that contains approximately 60 000 sixty-mer probes with 60 kb average probe spacing, with a higher resolution within the region recommended by International Standard Cytogenomic Array (ISCA) Consortium, was used. Slides were scanned on a SureScan Dx Microarray scanner (Agilent Technologies, USA) and processed with Feature Extraction software (v12.0). Results were analyzed using Agilent CytoGenomics (v3.0 and v4.0) software. The results were according to UCSC Human Genome build 19 (National Center for Biotechnology Information build 37) and included imbalances with at least three consecutive probes with abnormal log2 ratios. UCSC genome browser (http://genome.ucsc.edu), DECIPHER (DatabasE of Chromosomal Imbalances and Phenotypes using Ensembl Resources - http://decipher.sanger.ac.uk/), PubMed (http://www.ncbi.nlm.nih.gov/PubMed), ClinVar-NCBI (https://www.ncbi.nlm.nih.gov/clinvar), OMIM (Online Mendelian Inheritance in Man - http://www.ncbi.nlm.nih.gov/omim) and Database of Genome Variants (http://projects.tcag.ca) databases were used in the interpretation of the results.

CNVs were categorized as pathogenic, benign and VOUS based on well-established microdeletion or microduplication regions, inheritance pattern, gene content, and size as described elsewhere (1,3). The term VOUS was used when variants were less than 300 kb and when or if they included genes of unknown clinical significance and when family studies were inconclusive or unavailable.

## Results

In 73 patients, clinically relevant variants in the genome were detected and better characterized; there were 61 microdeletions and 30 microduplications (Table 1). In 19 patients, multiple imbalances were found (Table 2). Most of the pathogenic CNVs (68/91) were >1 Mb. VOUS were discovered in 35 patients: 25 had one VOUS, seven had two VOUS, and three subjects had one pathogenic CNV and one VOUS (subjects No. 49, 22, and 70). There were 17 microdeletions and 25 microduplications (Table 3). Only seven of these 42 microdeletions and microduplications were >1Mb.

**Table 1:** Pathogenic copy number variations (CNVs) involving a single genomic region*

Patient No.	ISCN description (2016)†	Size (kb)	Sex	Age at examination (years)	ID/DD	ASD	CA	Dysmorphism
99.	1p36.33p36.32(834101_2558913)x1	1724	F	6	x		x	x
5.	1q24.1-q25.1(166325047_176680992)x1 dn	10 356	M	5	x		x	x
8.	2p16.3(50886387_50947729)x1	61	F	6	x	x		
2.	2p16.3(50909765_50970721)x1	61	M	0 (1 month)	x		x	x
30.	2p25.3(1788489_1973174)x3	185	F	13	x			
19.	2q21.1(131501506_131915718)x1 mat	414	M	5	x	x		
51.	2q22.2q22.3(143931445_146479587)x1^‡^	2548	M	12	x		x	x
25.	3p25.3(10150872_10309577)x1	159	F	11	x		x	
48.	3q26.31(176573342_178552807)x1	1979	F	0 (6 month)	x		x	
73.	4p16.3(45882_686480)x3 pat	641	M	4	x			
61.	4p16.3(72447_2747165)x1^‡^	2675	F	8	x			x
17.	4p16.3p16.1(72447_8732731)x1 dn^‡^	8660	M	3	x	x	x	x
103.	5p13.2(36520866_37406919)x3 dn	886	F	5	x			x
96.	5p15.33p13.2(22149_35622770)x1 mat^‡^	35 601	F	3	x		x	x
63.	6p25.2p24.2(3090209_11327673)x3 mat	8237	M	16			x	x
68.	6q27(168682821_170921089)x1	2238	F	13	x		x	x
50.	7q11.23(72766313_74133332)x1^‡^	1367	M	1	x		x	x
71.	7q35(145815487_145934608)x1	119	M	11	x			
79.	8p22p21.3(13468338_23134996)x1	9667	M	7	x			x
57.	8p23.1(7169490_12404062)x1^‡^	5235	M	5	x		x	x
33.	8p23.3p11.1(191530_43529733)x3^‡^	43 338	F	6	x		x	x
38.	8q23.1q24.12(109937640_120021371)x1 dn^‡^	10 084	M	18	X		x	x
77.	8q23.3q24.11(117686699_118979648)x1^‡^	1293	M	19	x		x	x
62.	9p24.3p13.1(204193_38741437)x3	38 537	M	8	x		x	x
46.	9p24.3p22.2(204193_16705259)x1 dn^‡^	16 501	F	14	x		x	x
105.	9q34.3(140164421_141018984)x1^‡^	855	M	4	x		x	x
39.	10q11.22q11.23(48364954_51780909)x3	3416	M	6	x		x	x
15.	10q23.31q23.32(92855758_93832017)x3 mat	976	M	5	x			
40.	13q22.2q31.3(75400788_93036301)x1^‡^	17 636	M	3	x		x	x
92.	15q11.2(22765628_23217514)x1	451	M	2	x			
64.	15q11.2(22765628_23300287)x1	535	M	10	x			x
106.	15q11.2(22765628_23300287)x1	535	M	5			x	
85.	15q11.2q13.3(22765628_32899558)x3 dn	10 134	F	11	x	x		
3.	15q11.2q13.1(23656936_28520313)x1^‡^	4863	M	2	x			x
28.	15q11.2q13.1(23656936_28520313)x1	4863	F	17	x		x	x
60.	15q23q25.1(69058773_78855259)x1^‡^	9797	M	11	x		x	x
58.	15q26.2q26.3(96869390_102383473)x1^‡^	5514	F	7	x		x	
89.	15q26.3(99352805_102156616)x1^‡^	2804	F	9	x			x
80.	16p11.2(29673954_30190568)x3	517	F	10	x		x	x
13.	16q23.1q24.3(78387160_88755371)x1	10 368	M	12	x		x	
32.	17p11.2(16637902_20294038)x1 dn^‡^	3656	M	8	x	x	x	x
31.	17p12(14111772_15442066)x3 mat	1330	M	13			x	
1.	17p13.3p13.1(24457_6566906)x3	6542	F	6	x			
24.	17q21.31(43706886_44485830)x1	779	M	14	x		x	x
41.	17q24.1q24.3(62939944_68316019)x1	5376	F	1	x		x	x
47.	20q13.33(61704244_62908674)x1	1204	F	0 (1 month)	x			
45.	21q11.2q22.3(15485008_48090317)x3	32 605	M	6	x		x	x
67.	21q22.12q22.2(37635939_41718667)x3^‡^	4083	F	3	x		x	x
54.	22q11.21(18661724_21505417)x1	2844	M	0 (2 month)	x		x	x
37.	22q11.21(18661724_21505417)x1	2844	M	3	x		x	x
59.	22q11.21q11.22(21505358_22905068)x1^‡^	1400	M	3	x		x	x
34.	22q11.21(18706001_18984519)x3 mat	279	M	2	x	x		x
75.	22q13.31q13.33(46928208_51178264)x1^‡^	4250	F	3	x		x	x
69.	Xq28(153287517_153541289)x3 dn	254	F	2	x			

*Abbreviations: CNVs - copy number variations; ISCN - International System for Human Cytogenetic Nomenclature; kb - kilobases; ID/DD - intellectual disability/developmental delay; ASD - autism spectrum disorders; CA - congenital anomalies; F – female; M – male; dn - *de novo*; mat - maternally inherited; pat - paternally inherited.

†In the ISCN report, monosomy/deletion/one copy is designated with x1 and trisomy/duplication/three copy is designated with x3 in the genome.

‡The patients in whom chromosomal imbalances have already been detected by high resolution conventional cytogenetics, MLPA (Multiplex Ligation-dependent Probe Amplification) or FISH (Fluorescence In Situ Hybridization). The results are according to UCSC Human Genome build 19 (National Center for Biotechnology Information build 37).

**Table 2:** Pathogenic copy number variations (CNVs) involving two or more genomic regions*

Patient No.	ISCN description (2016)^†^	Size (kb)	Sex	Age at examination (years)	ID/DD	ASD	CA	Dysmorphia
20.	1p31.1(79322064_79541419)x1,22q11.21(18661724_21505417)x1	219/2844	M	3	x		x	x
56.	1p36.33p36.32(779727_5080691)x3,15q13.3(32065000_32509926)x3, 21q22.3(46346682_48084156)x1^‡^	4301 /445/1737	M	9	x		x	x
78.	1q21.1q21.2(146507518_147824207)x1,16p11.2(28843773_29031059)x1	1317/187	M	15	x		x	x
11.	2q32.3q33.1(193.987.239_199.889.760)x1 dn, 2q33.2q33.3(203583507_205732083)x1 dn, 2q34q35(209245201_216247586)x1 dn	5903/2149/7002	M	5	x		x	x
10.	2q37.3(239128062_243068396)x1 dn,9p24.3p13.3(204193_34206653)x3 dn^‡^	3940/34 002	M	1	x		x	x
9.	3p26.3p26.1(93949_6894668)x3 mat, 18q22.1q23(64900852_78012829)x1 mat^‡^	6801/13 112	F	18	x		x	x
14.	4p16.3p16.1(45882_8732731)x3 dn,8p23.3p23.1(191530_6880363)x1 dn^‡^	6689/8687	F	12	x	x		x
42.	4q32.2q35.2(162419540_190896674)x3 mat, 18q22.1q23(66312776_78012829)x1 mat^‡^	28 477/11700	F	3	x		x	x
91.	5p15.33(22149_2447692)x3 pat,18q22.1q23(61965606_78012829)x1 pat^‡^	2426/16 047	M	1			x	x
65.	5p15.33(22149_4163906)x1 mat,12p13.33p12.2(230421_20006466)x3 mat^‡^	4142/19 776	F	13	x		x	x
27.	5q14.1q14.2(78490881_81768020)x3 dn,17q12(34856055_36248918)x1 mat	3277/1393	M	3	x		x	x
49.	7q11.23(72766313_73735597)x1, *Xq28(154118643_154560375)x1*	969/442	F	3			x	x
83.	7q34q36.3(142328008_159124131)x1 mat, 18q23(76929981_ 78012829)x3 mat^‡^	16 796/1083	F	2	x		x	x
22.	*8p23.1(11384389_11586362)x3 mat*, 11p15.1p11.2(18826790_43592489)x1 dn	202/24 766	F	1	x		x	
53.	8p23.3(1224868_1513649)x3, 8p23.3(1554187_1628286)x1	289/74	F	4	x		x	
16.	8p23.3p23.1(191530_8.079920)x1 pat, 12p13.33p13.31(230421_8309723)x3 pat^‡^	7888/8079	M	10	x		x	x
6.	12p13.33p13.32(230421_4939008)x1 pat, 22q13.31q13.33(45277037_51178264)x3 pat^‡^	4709/ 5901	F	15	x		x	x
70.	17q12(34611352_36248918)x3, *18p11.31p11.23(6973010_805587)x3*	1638/1083	M	2	x		x	
55.	18p11.32p11.23(148963_7983966)x1 dn, 18q21.2q23(52625076_78010032)x1 dn	7835/25 385	M	4	x		x	x

*Abbreviations: CNVs - copy number variations; ISCN - International System for Human Cytogenetic Nomenclature; kb - kilobases; ID/DD - intellectual disability/developmental delay; ASD - autism spectrum disorders; CA - congenital anomalies; M – male; F – female; dn - *de novo*; mat - maternally inherited; pat - paternally inherited;.

†In the ISCN report, monosomy/deletion/one copy is designated with x1 and trisomy/duplication/three copy is designated with x3 in the genome.

‡The patients in whom chromosomal imbalances have already been detected by high resolution conventional cytogenetics, MLPA (Multiplex Ligation-dependent Probe Amplification) or FISH (Fluorescence In Situ Hybridization). In italic are designated variants of unknown clinical significance. The results are according to UCSC Human Genome build 19 (National Center for Biotechnology Information build 37).

**Table 3:** Variants of unknown clinical significance involving one or more genomic region*

Patient No.	ISCN description (2016) †	Size (kb)	Sex	Age at examination (years)	ID/DD	ASD	CA	Dysmorphia
36.	1p13.3(108332063_108739610)x3,16p13.3(3704209_3716095)x1	408/12	M	7	x	x		
87.	1q21.1(145415190_145799602)x3	384	F	11	x			x
86.	1q21.1(145415190_145799602)x3 pat,1q21.2(147381357_149768855)x3 pat	384/2387	M	7	x			x
98.	1q32.3q41(214316368_215890520)x1 dn	1574	M	8	x			x
74.	1q43(242196930_242442157)x1 pat	245	M	14	x			
97.	2q37.3(239970755_240317187)x3 Xq13.2q13.3(73039814_74232626)x2	346/1193	M	13	x		x	x
7.	3p26.3(2347184_2728530)x1 mat	381	F	3	x		x	x
21.	3p26.3(2649825_2660973)x3	11	F	1	x			
94.	3p26.3p26.2(2309008_2815363)x3	506	M	2	x			
52.	3q25.1(148990670_149767161)x1 dn,10q24.1(97230835_97546470)x1 mat	776/316	F	9	x			x
44.	3q28(191413759_191931899)x3 pat	518	F	6		x		x
81.	6q14.3(87229264_87662392)x3	433	F	3	x			
76.	7q32.3q33(131707914_133070269)x3	1362	M	3	x	x		
66.	9q22.31(95410466_96011338)x1	601	F	5	x		x	x
29.	10q23.1(84270008_84283542)x1, 22q11.22(23056562_23208022)x3	14/969	F	8	x		x	x
90.	12q24.13q24.21(114277899_114518222)x3 mat	240	F	1			x	x
93.	15q11.2(22765628_23300287)x3	535	M	1			x	
88.	15q13.3(32065000_32509926)x3 pat	445	M	8			x	x
35	15q26.3(100569135_102383473)x1 mat	1814	F	5	x	x		x
23.	16p13.11(14910205_16194578)x3 pat	1676	F	5		x		
4.	16p13.3(6991421_7036068)x1	45	M	2	x	x		
100.	16q23.1(77351997_78187104)x3	835	M	1	x		x	x
95.	21q22.3 (45814926_46505455)x3	691	M	8	x			x
26.	22q11.23(23895563_24178173)x3	283	F	8	x		x	x
12.	Xp11.23(47330212_47335227)x0 mat, Xq13.3(74463757_74651249)x2 mat	5/187	M	7	x		x	x
18.	Xp11.23(47330212_47335227)x0	5	M	3	x			
84.	Xp11.23(47330212_47335227)x0 mat	5	M	9	x		x	x
101.	Xp22.2(13945712_14167313)x0 dn,4q22.3(98501338_98757811)x1 mat	221/256	M	10	x		x	x
72.	Xp22.33(3313941_3911921)x1 dn	598	F	1	x			
82.	Xq21.1(77105411_77127453)x2	22	M	21			x	
104.	Xq21.1(79777911_79932626)x2	155	M	3	x	x		
102.	Xq26.2(130674304_130950243)x2 mat	276	M	3	x	x		x

*Abbreviations: ISCN - International System for Human Cytogenetic Nomenclature; kb - kilobases; ID/DD - intellectual disability/developmental delay; ASD - autism spectrum disorders; CA - congenital anomalies; M – male; F – female; dn - *de novo*; mat - maternally inherited; pat - paternally inherited.

†In the ISCN report, monosomy/deletion/one copy is designated with *x1*, trisomy/duplication/three copy is designated with *x3* in the genome, with *x0* deletion on X chromosome in a male subject, and with *x2* duplication on X chromosome in a male subject. The results are according to UCSC Human Genome build 19 (National Center for Biotechnology Information build 37).

In the ID/DD, ASD, CA with/without dysmorphism group, there were only two patients with pathogenic CNVs. In the ID/DD, CA with/without dysmorphism group, there were 47 subjects with pathogenic CNVs and nine with VOUS. In the ID/DD with or without dysmorphism group, five patients had pathogenic CNVs and six had VOUS. In the ID/DD, ASD with or without dysmorphism group, there were 14 patients with pathogenic CNVs and 11 with VOUS. There were five patients with pathogenic CNVs and four with VOUS in the CA with/without dysmorphism group, and only two patients with VOUS in the ASD with/without dysmorphism group.

The distribution of 51 newly diagnosed pathogenic CNVs and 42 VOUS by size showed that there were no VOUS greater than 5 Mb (Table 4). The smallest CNV was hemizygous deletion of 5 kb categorized as VOUS that partially encompasses *ZNF41* gene in Xp11.23 region detected in three male patients. The largest imbalance was the duplication of 43 338 kb and included a whole 8p arm. A high resolution cytogenetic analysis revealed mosaic tetrasomy 8p: 47,XX,i [8](p10)[18]/46,XX[82].

**Table 4:** Size distribution of pathogenic copy number variations (CNVs) and variants of unknown clinical significance (VOUS) found in patients

Size of imbalance	No. (%) pathogenic CNVs	No. (%) of VOUS
>10 Mb	7 (13.7)	0
5-10 Mb	7 (13.7)	0
1-5 Mb	16 (31.4)	7 (16.7)
1 Mb-500 kb	8 (15.7)	9 (21.4)
500-300 kb	2 (3.9)	9 (21.4)
<300 kb	11 (21.6)	17 (40.5)
Total	51 (100.0)	42 (100.0)

Genomic regions that were the most commonly affected by pathogenic CNVs were 8p23 (6 patients), 15q11.2 (6 patients), and 22q11.21 (5 patients). In the group with complex rearrangement, the most affected, relatively large region was 18q22.1q23 (5 patients).

Parents of 44 probands with pathogenic CNV or VOUS were available for DNA testing. Four VOUS were *de novo* and 16 were inherited. In eight cases, pathogenic CNVs were inherited from parents with balanced translocations, in seven cases one of the parent had same pathogenic CNV as his offspring, and 19 pathogenic CNVs were *de novo.*

## Discussion

Pathogenic CNV were found in 43 of total 307 patients previously not tested by other cytogenetic or molecular methods or in whom these tests were negative for genomic imbalances, representing an overall diagnostic yield of 14%. Our results are in accordance with other studies on genome-wide oligonucleotide arrays (1,4,5). The diagnostic yield is known to correlate with the array resolution and the genomic coverage of the array used. Currently, we are using 8x60K array with 60 kb overall median probe spacing (higher in ISCA regions), and the results of this study showed that this platform is suitable for genetic testing of children with developmental disorders.

The microdeletion syndromes that were identified in more than one individual were: 15q11.2 deletion syndrome (MIM#615656)-3 patients, 22q11.21 deletion syndrome (MIM#188400; DiGeorge syndrome)-3 patients, 4p16.3 deletion syndrome (MIM#194190; Wolf-Hirschhorn syndrome)-2 patients, 7q11.23 deletion syndrome (MIM#194050; Williams-Beuren syndrome)-2 patients, 8q24.1 deletion syndrome (MIM#150230; Trichorhinophalangeal syndrome, type II)- 2 patients, 15q11.2q13.1 deletion (MIM#105830; Angelman syndrome/MIM#176270; Prader-Willi syndrome)-2 patients, and 15q26-qter deletion syndrome (MIM#612626))-2 patients. Also, the same 61 kb deletion in 2p16.3 region disrupting *NRXN1* gene was detected in two patients.

One third of pathogenic CNVs ranged 1-5 Mb in size. Even though CNVs of <300 kb presented 21.6% of pathogenic CNVs, CNVs of this size were almost twice less presented in the category of pathogenic CNVs as compared to VOUS category. In pathogenic CNV category, there were twice more deletions than duplications, whereas microduplications were more frequent in VOUS category.

VOUS were reported in 35 of 307 cases, including three cases with additional pathogenic CNV. The sizes of the VOUS varied from 2387 kb to 5 kb. The largest VOUS was duplication in the 1q21.2 region inherited from the apparently healthy father. The duplication involved only one pathogenic gene (*GJA8*) and partly affected the region causing 1q21.1 duplication syndrome (#612475 MIM). It is considered that a critical region causing 1q21.1 duplication syndrome is 800 kb in size (chr1: 146577487-147394506 GRCh37/ hg19) and includes at least 7 genes (6). Duplication in our patient overlapped only in 13 kb, and included only *GJA8* gene associated with cataract not present in the patient. Hence, although the duplication was relatively large, it was classified as VOUS based on the gene content and the fact that it was inherited from the apparently healthy father. The smallest CNVs on X chromosome detected in three male patients were inherited from the normal heterozygous mother in patients No.s 12 and 84. The most common size category of VOUS was <300 kb (40.5%). Based on the clinical presentation of our patients, family studies, type of CNV (deletion vs duplication), gene content, and the size distribution of pathogenic CNVs and VOUS, we recommend using a 300 kb as an arbitrary cut off for clinically relevant CNV when using this platform.

The inheritance pattern of a CNV, when accompanied by clinical and family history information, can be useful. However, in the clinical setting can sometimes be difficult to test both parents. *De novo* CNVs are more likely than inherited CNVs to be pathogenic. In this study, of 15 *de novo* CNVs, 13 were pathogenic and only two were VOUS.

The inherited CNVs may cause a range of severity of clinical presentation. In four of seven cases where one of the parent had the same pathogenic CNV as offspring, parents were phenotypically normal (patients No. 19, 73, 15, and 34), in two cases clinical presentation was milder (patients No. 63 and 27) and in one case the mother had same phenotype (patient No. 31). This can be exemplified by case 63, where the male patient inherited dup 6p25.2p24.2 from his mother. Distal 6p trisomy is very rare. Patients have variable clinical findings with duplications that usually range from 6pter to 6p21-6p25 (7). The phenotypic features that are associated with dup 6p25.2p24.2 that were present in our patient were tall stature, dysmorphia, obesity, frequent respiratory infections, foot malformations, hypoplastic left kidney, hypospadias, and urethral stenosis. His 37-year old mother was dysmorphic, obese like her son, but had no associated anomalies. In addition, she suffered from osteopetrosis and polyarthralgia, which are presumably of different etiology. Despite the large duplications, both had completely normal intellectual functioning.

The CNVs that contain many genes or known disease genes are more likely to be pathogenic than those that contain few genes or genes of uncertain function. Thus, large CNVs are more likely than small CNVs to cause clinical manifestations as they generally encompass more genes, with a higher probability to affect a dosage-sensitive one. As deletions result in haploinsufficiency, some very small deletions, for example 61 kb deletion in 2p16.3 region altering *NRXN1* gene (patients No. 8 and 2), can also be pathogenic (8). Duplications are more difficult to interpret because some relatively large duplications have no pathogenic effect and are found in normal subjects. CNVs within regulatory regions of clinically relevant genes make interpretation even more complex. In the patient No. 11, CMA analysis found three consecutive deletions in region 2q32.3q35. The analysis of subtelomeric regions and microdeletion syndromes by MLPA method was negative. Most of his phenotypic features overlapped with the clinical presentation of 2q33.1 syndrome (MIM # 612313, Glass syndrome). The gene *SATB2* was not directly affected by the deletion in region 2q33.1. For this reason, the MLPA analysis using P245-B1 kit targeting within the 2q33.1 region only gene *SATB2* was negative. The first deletion of 2q32.3-33.1 had a distal break point between genes *PLCL1* and *SATB2*. This probably led to a disruption of *SOX9-* mediated cis-regulation resulting in functional haploinsufficiency of *SATB2* (9).

The retrospective study of 30 patients by CMA analysis successfully confirmed all previously detected genomic changes. The genomic breakpoints and the gene content were defined, allowing for more precise genotype-phenotype delineation. Furthermore, some additional changes were found in patient No. 56 in whom 15q13.3 duplication of 445 kb was not detected by MLPA, and in patients No.s 83 and 91, in whom 18q23 duplication of 1083 kb and 5p15.33 duplication of 2426 kb, respectively, were missed by chromosome karyotyping. This discordance may be explained by targeted analysis of the genome provided by MLPA probes and a small resolution of the conventional karyotyping, usually 5-10 Mb.

The major difference between patients with pathogenic CNVs and patients with VOUS was present in groups with ID/DD, ASD, CA with/without dysmorphism; ID/DD, CA with/without dysmorphism; and ASD with/without dysmorphism. This was expected, considering the size and number of pathogenic genes encompassed by pathogenic CNVs. ASD with/without dysmorphism was present in seven patients with pathogenic CNVs only in combination with ID/DD and/or CA and in the VOUS group, it was twice as frequent as in the group with pathogenic CNVs. Subsets of individuals with ASD are more likely to carry disruptive *de novo* and rare CNVs and sequence-level mutations (10). Microarray testing identifies etiology of ASD in 8%-21% of cases. Overall, CMA may identify a clinically significant CNV in about 10% of all cases of ASD (11). Whole exome sequencing (WES) and whole genome sequencing (WGS) are expected to increase significantly the diagnostic yield when applied to patients with ASD. A recent report suggests that WES/WGS may identify an additional 10%–15% of causes of ASD. Together WES and CMA may identify the cause of ASD in 20% of cases (12). Hence, further testing of ASD subjects with next generation sequencing technique is the next step in our genetic testing protocol.

In summary, our results showed that Agilent ISCA v2 Human Genome 8x60 K oligonucleotide microarray format provided a reasonable resolution for clinical use, particularly in the ISCA regions containing known disease genes associated with well-established phenotypes. The CMA method revised the MLPA and conventional karyotyping results and provided a new, more detailed insight into genomic changes. It is to be expected that increasing number of smaller pathogenic CNVs will be discovered because there is a tendency for an increasing number of laboratories to use CMA platforms of higher resolution. This will simultaneously lead to an increased number of VOUS and the need to include other criteria for establishing their significance, based on data collection on new patients, genotype-phenotype correlation, and better understanding of the complex interaction of the genes included in the CNVs with the entire genome.

## References

[1] Miller DT, Adam MP, Aradhya S, Biesecker LG, Brothman AR, Carter NP (2010). Consensus statement: chromosomal microarray is a first-tier clinical diagnostic test for individuals with developmental disabilities or congenital anomalies.. Am J Hum Genet.

[2] Cooper GM, Coe BP, Girirajan S, Rosenfeld JA, Vu TH, Baker C (2011). A copy number variation morbidity map of developmental delay.. Nat Genet.

[3] Lee C, Iafrate AJ, Brothman AR (2007). Copy number variations and clinical cytogenetic diagnosis of constitutional disorders.. Nat Genet.

[4] Sagoo GS, Butterworth AS, Sanderson S, Shaw-Smith C, Higgins JP, Burton H (2009). Array CGH in patients with learning disability (mental retardation) and congenital anomalies: updated systematic review and meta-analysis of 19 studies and 13,926 subjects.. Genet Med.

[5] Xiang B, Zhu H, Shen Y, Miller DT, Lu K, Hu X (2010). Genome-wide oligonucleotide array comparative genomic hybridization for etiological diagnosis of mental retardation: a multicenter experience of 1499 clinical cases.. J Mol Diagn.

[6] Bernier R, Steinman KJ, Reilly B, Wallace AS, Sherr EH, Pojman N (2016). Clinical phenotype of the recurrent 1q21.1 copy-number variant.. Genet Med.

[7] Jankauskienė A, Koczkowska M, Bjerre A, Bernaciak J, Schaefer F, Lipska-Ziętkiewicz BS (2016). Glomerulopathy in patients with distal duplication of chromosome 6p.. BMC Nephrol.

[8] Dabell MP, Rosenfeld JA, Bader P, Escobar LF, El-Khechen D, Vallee SE (2013). Investigation of NRXN1 deletions: clinical and molecular characterization.. Am J Med Genet A.

[9] Rainger JK, Bhatia S, Bengani H, Gautier P, Rainger J, Pearson M (2014). Disruption of *SATB2* or its long-range cis-regulation by SOX9 causes a syndromic form of Pierre Robin sequence.. Hum Mol Genet.

[10] Iossifov I, O’Roak BJ, Sanders SJ, Ronemus M, Krumm N, Levy D (2014). The contribution of de novo coding mutations to autism spectrum disorder.. Nature.

[11] Schaefer GB, Mendelsohn NJ (2013). Professional Practice Guideline Committee Clinical genetics evaluation in identifying the etiology of autism spectrum disorders: 2013 Guideline revisions.. Genet Med.

[12] Tammimies K, Marshall CR, Walker S, Kaur G, Thiruvahindrapuram B, Lionel AC (2015). Molecular diagnostic yield of chromosomal microarray analysis and whole-exome sequencing in children with Autism Spectrum Disorder.. JAMA.

